# Characterization of the influence of the dominant tract on hand closing post stroke based on the Fugl-Meyer score

**DOI:** 10.1038/s41598-023-28290-z

**Published:** 2023-02-14

**Authors:** Raziyeh Baghi, Dongwon Kim, Kyung Koh, Li-Qun Zhang

**Affiliations:** 1 Department of Physical Therapy and Rehabilitation Science, University of Maryland, Baltimore, MD USA; 2 EpicWide, LLC, Baltimore, USA; 3 Department of Orthopedics, University of Maryland, Baltimore, MD USA; 4 Department of Bioengineering, University of Maryland, College Park, MD USA

**Keywords:** Neuroscience, Physiology, Neurology

## Abstract

While stroke survivors with moderate or mild impairment are typically able to open their hand at will, those with severe impairment cannot. Abnormal synergies govern the arm and hand in stoke survivors with severe impairment, so hand opening, which is required to overcome the working synergy, is an extremely difficult task for them to achieve. It is universally accepted that alternative tracts including the cortico-reticulospinal tract (CRST), employed in the case that the corticospinal tract (CST) is damaged by stroke, brings about such abnormal synergies. Here we note that hand closing is enabled by alternative tracts as well as the CST, and a research question arises: Does motor characteristics while closing the hand depend on the integrity of the CST? In this study, we evaluate the abilities of 17 stroke survivors to flex and relax the metacarpophalangeal (MCP) joints and investigate whether motor characteristics can be distinguished based on CST integrity which is estimated using upper-extremity Fugl-Meyer (UEFM) scores. UEFM scores have been perceived as an indirect indicator of CST integrity. We found that participants with the UEFM score above a certain value, who are assumed to use the CST, moves the MCP joints more smoothly (*P* < 0.05) and activates the flexors to flex the joints faster (*P* < 0.05), in comparison to participants with low UEFM scores, who are assumed to preferentially use alternative tracts. The results imply that use of alternative tracts (i.e. the CRST) results in a degradation in movement smoothness and slow activation of MCP flexors. We present evidence that responses of flexors of the MCP joints following stroke depend on the degree of impairment which is hypothesized to originate from preferentially use of different neural motor pathways.

## Introduction

Hand dexterity is substantially influenced by synergies caused by stroke^1–5^. Hand opening and closing, even this simple motion is possible only if the motor drive to the appropriate joints of the hand needs to overwhelm the synergy that is dominant over the hand^4^. Originally, synergy is a concept that describes low-dimensional movement expressed in a higher dimensional space of possible muscle activations. Stroke creates abnormal synergies through regrouping muscle groups that are innervated together in the context of executing movements. It is typical that stroke re-groups muscle groups and causes abnormal couplings among the muscle groups across the limbs. The so-called flexion synergy and extension synergy are representative stroke-caused characteristics in the affected individuals and involve abnormal co-activation of shoulder abductor and adductor with distal limb flexor and extensor, respectively^6–8^.

Such pathological synergies largely originate from damage to the corticospinal tract (CST). As an adaptive strategy, the reliance on cortico-bulbospinal motor pathways increases that are closely related to flexion and extension synergy expressions. There is extensive evidence that the cortico-reticulospinal tract (CRST) especially enhances flexion synergy expression, among cortico-bulbospinal motor pathways including the CSRT and the cortico-rubrospinal tract (CRBT)^9–13^. Diffusor tensor imaging (DTI) studies showed the inverse relationship between CST integrity and CRST integrity^10,14,15^. Several studies demonstrate that the influence of the CRBT is relatively low in humans^10,16^. Generally we observe that while use of alternative tracts leads to stroke-caused synergies, the CST enables motion out of those synergies^13^. When alternative tracts are assumed to be activated, monotonous synergistic movement is dominant^12,17^. It is very difficult for stroke survivors who are unable to employ the CST to perform a movement out of synergy. Executing hand opening and closing with an arm posture under a working synergy may be a difficult task when the stroke survivor preferentially uses alternative tracts other than the CST^4^. The evidence that involuntary grasping is in correlation with increased activation in contralesional cortical areas, not ipsilesional cortical areas, implies that hand movements are governed by the CRST^12^.

Here, a question arises as to whether there is a difference in the ability to flex the fingers according to the usabilities of the CST and its alternative tracts. Regardless of whether the CST is in use, stroke survivors are seamlessly capable of closing their hands. There is evidence that use of the CST is generally dominant all the time after the onset of stroke if the initial CST integrity is retrievable^18–20^. Meanwhile it is generally assumed that use of alternative tracts is dominant all the time if the initial CST integrity is poor. We might divide individuals with stroke into two groups; one preferentially uses the CST, the other preferentialy uses alternative tracts.

Upper-extremity Fugl-Meyer (UEFM) scores have been recognized as an indirect indicator of the dominance of tracts. Though there surely lacks clarification on ﻿a dividing line of the UEFM total score, it is reasonably speculated that a greater UEFM total score implies a higher possibility that use of the CST is dominant over any other tracts. The proportional recovery rule that describes the recovery of an average of 70% between the onset of stroke and the starting point of the chronic phase is based on UEFM scores^19–21^. While this rule holds true for patients across all ages and sexes^21,22^, breakers of this rule (so-called non-fitters) are identified as patients who lack CST integrity. Fitters who are normally perceived to have CST integrity enter the chronic phase with the UEFM total score of 40 or above, while non-fitters do not typically reach 40^20,22^. A recent study based on UEFM scores showed that the CST begins to be used in persons with stroke in the chronic phase roughly with a UEFM total score of 30 or above^4^.

In this study, we investigate timings in muscle activation as well as in deactivation in participants with stroke hemiparesis in the late sub-acute or chronic stage during a finger-flexing task. Participants are requested to flex and relax the metacarpophalangeal (MCP) joints against motorized resistance in response to audible tones. Movement smoothness of the MCP joints is also evaluated that could be an indicator of muscle individuation to distinguish a motor execution feature of the CST versus its alternatives. We strive to find conspicuous differences in measures for flexing motion originating from the usabilities of the CST or its alternatives based on a UEFM score. We hypothesize that there might be a significant difference in motor execution between two groups who are divided based on the usability of the CST.

## Methods

### Participants

17 hemiplegic volunteers post stroke (age: 54.31 ± 13.10 (S.D) years; sex (F/M): 4/13; impaired side (L/R): 9/8, time since stroke: 7.63 ± 6.51 (S.D) months) participated in the study. The inclusion criteria were moderate-to-severe upper extremity impairment (UEFM score < 50) and sufficient cognitive/language abilities to follow instructions during the experiment (Mini-Mental Status Score > 22). We excluded volunteers who had severe shoulder pain, relevant musculoskeletal injury, or fixed contraction deformity in the upper extremity. None of the participants received pharmacological medications for spasticity and tone (i.e. Botulinum toxin injection to the upper limb) in the 5 months before the experiment. Each participant gave written informed consent. The experimental protocol was approved by the Institutional Review Board of the University of Maryland, Baltimore. All methods were performed in accordance with the relevant guidelines and regulations.

### Procedure

Participants sat on a height-adjustable chair with a back support and their paretic hand was placed on a rotatable rigid plate that was connected to an electrical motor with an encoder (Maxon Brushless EC60, Sachseln, Switzerland). The MCP joints of the digits II-V were aligned with the rotation axis of the plate. The hand and forearm were supported being tightly fixed to the device using rigid mechanical blocks with cushion and Velcro straps. The arm posture was maintained with shoulder adduction of 45°, shoulder flexion of 45°, elbow flexion of 90°, and wrist flexion of 0°, respectively. Wireless EMG electrodes (Delsys, Boston, MA) were placed on the flexor digitorum superficialis (FDS) and extensor digitorum superficialis (EDS) muscles.

The experiment consisted of two sessions. The first session was to evaluate the ability of participants to flex and extend the MCP joints voluntarily. We asked them to move their MCP joints back and forth as much as possible. The hand plate was backdriveable while it measured the angle of the MCP joints of the digits II-V. The second session was to determine timing and muscle activity during MCP flexion. The MCP joints were passively locked by the motorized resistance when the joints were in the neutral position. Participants were requested to flex and relax the MCP joints against motorized resistance in response to audible tones. Three pairs of tones were given. Participants were asked to flex maximally, as quickly as possible, in response to the first tone of each pair, and relax as quickly as possible after the second tone. These pairs were placed 20 s apart to alleviate fatigue, and the first and second tones of the three pairs were gapped by 3, 2 and 4 s, respectively, to reduce the learning effect on timing. A practice period was given to each participant to get familiar with the instruction.

Data acquisition of joint angle and EMG signals were conducted in a LabVIEW environment. The sampling rate was set at 1000 Hz.

### Analysis

We conduct non-parametric correlation analyses (Spearman's rank correlation) of UEFM scores with the smoothness of MCP joint movement and delays in initiation and termination.

#### Smoothness of MCP joint movement

The spectral arc length is employed to evaluate the smoothness of the movement of the MCP joints^23,24^. Computation of the spectral arc length is carried out based on the angular velocity profile of the MCP joints $\omega_{MCP} \left( t \right)$ as

$$\begin{aligned} & \eta_{sal} \triangleq - \mathop \smallint \limits_{0}^{{\omega_{c} }} \sqrt {\left( {\frac{1}{{\omega_{c} }}} \right)^{2} + \left( {\frac{{d\hat{W}_{MCP} \left( \omega \right)}}{d\omega }} \right)^{2} } d\omega , \\ & \hat{W}_{MCP} \left( \omega \right) \triangleq \frac{{\hat{W}_{MCP} \left( \omega \right)}}{{\hat{W}_{MCP} \left( 0 \right)}}, \\ \end{aligned}$$

where $\hat{W}_{MCP}$(ω) is the Fourier magnitude spectrum of $\omega_{MCP} \left( t \right)$. and [0, $\omega_{c}$] is the frequency band where active movement is considered to occur.

In this study, $\omega_{c}$ is set at 4π rad/s, or 2 Hz, which covers active movements of the participants in this study. The angular velocity of the MCP joints is obtained through numerical differentiation of the angle of the MCP joints and zero-phase filtering with a 4th-order, 2-Hz Butterworth low-pass filter.

#### Delays in initiation and termination

**Figure 1:**
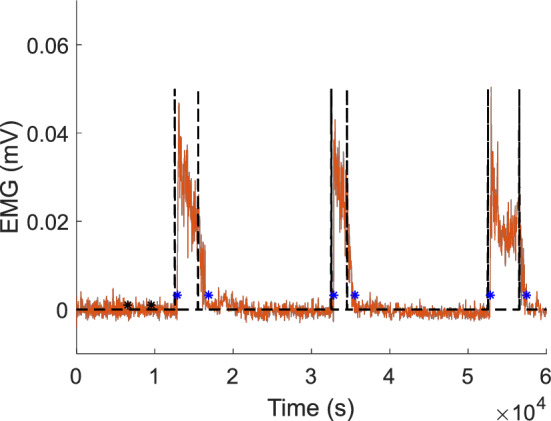
EMG response of a representative subject. Black dashed lines indicate the times points of audible tones occurring. The blue asterisks indicate the time points that are registered by responses of the subject versus a predefined threshold. The predefined threshold (3 standard deviations above the mean of EMG during the rest period) is based on the rest period defined by the black asterisks.

The delays are determined by the time gap between the occurrences of the audible tones and initiation/termination. Delays in initiation and termination of FDS and EDS are evaluated using EMG responses versus a predefined threshold (3 standard deviations above the mean of EMG during the rest period)^25^, as seen in Fig. 1. EMG signals are low-pass filtered at 225 Hz, rectified and low-pass filtered at 10 Hz using MATLAB (MathWorks, Natick, MA) to produce linear envelopes (LEs)^25^. EMG LEs are subtracted by the mean of the EMG LE during the rest period.

We average the values of delays across the three trials of flexion. Data which termination occurred before the tone are excluded from analysis.

In the case that we observe the possibility that our measures divide participants into two groups, we employ multivariate analysis of variance (MANOVA) with Bonferroni corrections to test whether there exist differences in movement features conveyed by the CST and its alternative tracts in terms of movement smoothness, and delays in initiation and termination of the flexor and extensor, with group as a between-subject variable. The significance level α is set as 0.05.

## Results

**Figure 2:**
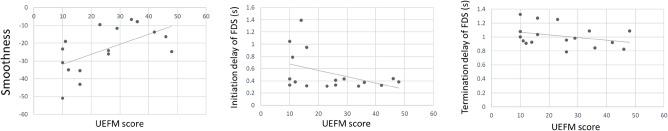
Relationships of the total UEFM scores with the smoothness of MCP joint movement and delays of FDS activation and de-activation. The data of one subject is missing for the smoothness results.

The correlation analysis reveals a significant positive correlation between movement smoothness and the total UEFM score (*P* = 0.027, r = 0.551), whereas other correlations are not statistically significant (*P* > 0.05), as presented in Table 1 and Fig. 2. The results indicate that as the total UEFM score increases, movement smoothness is generally better, while we observe no evidence that delays in initiation and termination of FDS and EDS correlate with the total UEFM score in our data.

**Table 1:** Results (Spearman's rank correlation coefficient and *p*-value) of correlation analysis of the total UEFM score with the smoothness of MCP joint movement and delays in initiation and termination.

	Smoothness	Initiation of FDS	Termination of FDS	Initiation of EDS	Termination of EDS
UEFM	r = 0.551, *P* = 0.027	r = − 0.336, *P* = 0.188	r = − 0.303, *P* = 0.238	r = − 0.196, *P* = 0.452	r = − 0.080, *P* = 0.760

**Figure 3:**
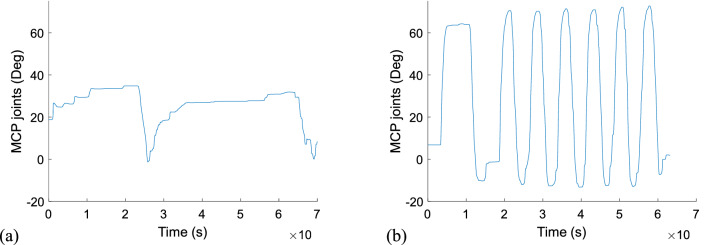
MCP joint movements of two representative subjects: (**a**) one (UEFM: 16) who would be assumed to preferentially use the CRST and (2) one (UEFM: 23) who would be assumed to preferentially use the CST.

**Figure 4:**
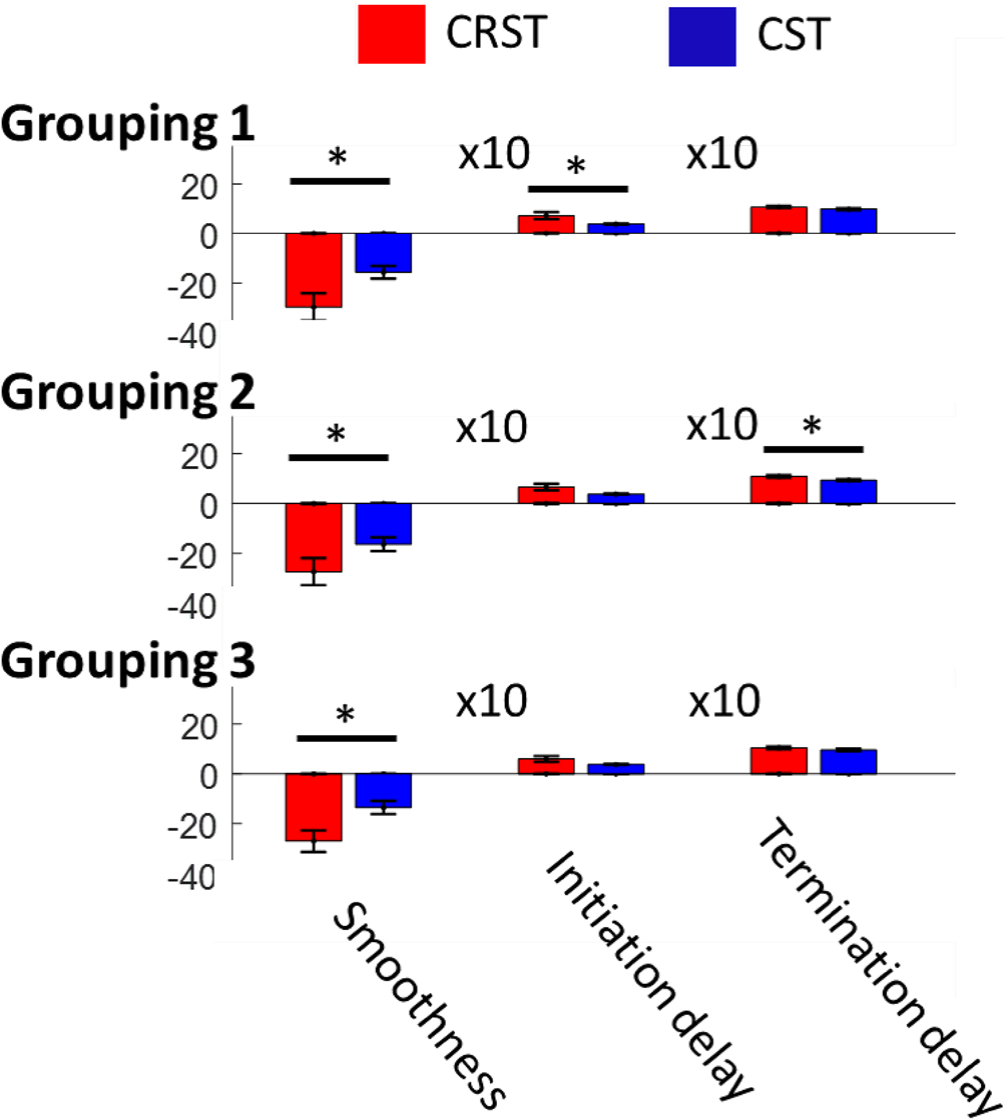
Statistical results of comparison between the CRST and CST groups varying grouping: smoothness of MCP joint movement and delays in initiation and termination of FDS. Grouping 1: subjects with UEFM 23 or above are in the CST group, Grouping 2: subjects with UEFM 26 or above are in the CST group, and Grouping 3: subjects with UEFM 29 or above are in the CST group. Error bars are ± 1 standard error of the mean.

We characterize the working features of the CST and CRST by grouping participants into two groups based on the total UEFM score in a binary manner^4^: the CST group (higher total UEFM score), who would be assumed to preferentially use the CST, and the CRST group (lower total UEFM score), who would be assumed to preferentially employ the CRST. From Fig. 2, we observe a clear dividing line with which we can divide participants into two groups based on the total UEFM score, that is, 20 (Grouping 1). Beyond the total UEFM score 20, movement smooth is better across corresponding participants, with a smaller variance (*P* < 0.05, F-test), in comparison to movement smooth of participants below the total UEFM score 20, which would imply that different motor pathways are preferentially used, assuming that preferential use of the CST leads to homoogeneity while preferential use of alternative tracts leads to heterogeneity). Indeed, as shown in Fig. 3, partipants with the total UEFM score 23 or above are capable of well extending their MCP joints, whereas partipants with the total UEFM score 16 or below are not capable; they make discrete-wise movements. MANOVA reveals significant differences in movement smoothness (*P* = 0.001) and delay in initiation of FDS (*P* = 0.039). The results imply that 9 subjects with the total UEFM score 23 or above have better movement smoothness and shorter delays in initiation of FDS, in comparison with 8 subjects with the total UEFM score 16 or below. In Grouping 2 with a threshold of the total UEFM score 23, significant differences are found in movement smoothness (*P* = 0.019) and delay in termination of FDS (*P* = 0.031). The results indicate that the CST group (8 subjects with UEFM 26-48) has better movement smoothness and shorter delays in termination of FDS, in comparison with the CRST group (9 subjects with UEFM 10–23). In Grouping 3 with the threshold of the total UEFM score 26, a significant difference is obeserved in only movement smoothness (*P* = 0.009). The results imply that the CST group (6 sunjects with UEFM 29-48) shows better movement smoothness, in comparison to the CRST group (11 subjects with UEFM 10-26). No other Groupings have significant differences (*P* > 0.05). The results were presented in Fig. 4 and Table 2.

**Table 2:** Mean ± 1 SD of smoothness of MCP joint movement and delays in initiation and termination of FDS and EDS, based on grouping.

	Smoothness	Initiation of FDS (s)	Termination of FDS (s)	Initiation of EDS (s)	Termination of EDS (s)
Grouping 1					
CRST	− 33.89 ± 10.97	0.70 ± 0.40	1.06 ± 0.16	1.12 ± 0.85	1.73 ± 0.84
CST	− 15.63 ± 7.59	0.37 ± 0.05	0.97 ± 0.15	0.86 ± 0.92	2.05 ± 0.71
Grouping 2					
CRST	− 30.84 ± 13.33	0.66 ± 0.40	1.08 ± 0.16	1.11 ± 0.79	1.74 ± 0.79
CST	− 16.40 ± 7.73	0.37 ± 0.05	0.94 ± 0.11	0.83 ± 0.98	2.08 ± 0.75
Grouping 3					
CRST	− 29.68 ± 12.01	0.61 ± 0.37	1.04 ± 0.17	0.98 ± 0.77	1.89 ± 0.80
CST	− 13.50 ± 6.57	0.38 ± 0.05	0.96 ± 0.11	0.98 ± 1.12	1.91 ± 0.77

Grouping 1: subjects with UEFM 23 or above are in the CST group, Grouping 2: subjects with UEFM 26 or above are in the CST group, and Grouping 3: subjects with UEFM 29 or above are in the CST group.

## Discussion

It is well accepted that the CST facilitates use of individual joints or muscles^13^, while alternative tracts including the CRST lead to synergistic movements in an abnormal manner^12^. It is assumed that after the CST recovers to be preferentially used, the stroke survivor retrieves hand functions^4^. In this study, we evaluated the ability of stroke survivors to move the MCP joints with smoothness and flex the MCP joints in response to cues and examined if the UEFM score can be an indicator.

Movement smoothness is a characteristic of motor coordination of stroke patients that covers the activation timings of the muscles in the antagonistic setup as well as the activation strength of the muscles. Movements of stroke patients generally regain smoothness with recovery^23,26^. We revealed that patients with greater UEFM scores look smooth sinusoidal motions at the MCP joints and they made more numbers of back-and-forth movements within an equivalent duration, in comparison to patients with lower UEFM scores (refer to Fig. 3). Regain of movement smoothness might imply that persons with stroke retrieve controllability of individual muscles. Also controllability of individual muscles allow them to make movements out of stroke-caused synergies (i.e. flexion synergy) if those exist. The affected arm posture in our experimental setup (shoulder adduction of 45°, shoulder flexion of 45°, elbow flexion of 90°) might cause abnormal synergy (we observed that several participants involuntarily activated muscles other than wrist muscles during the experiment even though their arm was at neutral), and extension of the MCP joints is possible only when the stroke survivor overcomes the synergy governed across the impaired upper extremity. We observed the difficulty of MCP extension in stroke patients with lower UEFM scores (i.e. < 16). It is reasonable to regard that stroke subjects with fine movement smoothness possess mature CST integrity.

Our grouping analysis revealed that the group with UEFM 23 or above, who would be assumed to preferentially use the CST, generated smoother movements at the MCP joints in comparison to the group with UEFM 16 or below, who would be assumed to preferentially use alternative tracts (i.e. CRST) (*P* < 0.05). Participants with UEFM 23 or above, who showed smoother movements, tended to have a faster response in the flexor. A statistically shorter delay in flexor activation in those participants would indicate preferential use of the CST . The results imply that the CST allows the flexor to activate in a faster way than alternative tracts including the CRST. In a previous study^25^, delay in grip initiation is greater for the paretic hand than for the non-paretic hand. Though the subjects in that study could preferentially use either the CST or CRST for the paretic hand, their results would be thought to be generally in agreement with our results regarding delay in initiation; we assume that participants in that study who may preferentially use the CRST showed an increased averaged delay of the paretic hand versus that of the non-paretic hand. For delay in termination, delay tends to be smaller as the total UEFM score increases, though it does not reach the statistical significance in the correlation analysis (*P* = 0.083). Hence, their results would be thought to be in agreement with the results presented in the previous study^25,27^. We generally speculate that delays in initiation and termination might originate from recruitment of muscles that are grouped by an abnormal synergy, and motor command to the abnormal synergy is conveyed through the CRST. If the strength of the synergy decreases, delays in muscle activation or/and de-activation would decrease accordingly. Indeed it was reported that delay in grip termination decreases with arm support^25^. Arm support is considered to contribute to a reduction in the flexion synergy^28,29^.

This study presented indirect evidence regarding the dominance of tracts ﻿based on UEFM scores, without support of a neurophysiological study. However our hypothesis and test are grounded in the general consensus about uses of the CST and its alternative tracts versus UEFM scores. There are inconclusive opinions as to whether tracts are used in a shared manner or exclusive manner. The Baker group has argued that hand gross movement is executed by the CRST as well as the CST, especially in the case of tasks that require grasping with strength^13,30^, while CST integrity is more influential on force generating with the fingers^18^. Leaving this controversy to future studies, we assume that it is strictly clear that dominance of tracts varies with the total UEFM score; the CST is dominant in patients with the range of high UEFM scores while the CRST is dominant in patients with the range of low UEFM scores. Neurophysiological studies showed that without mature CST integrity, hand functions are not fully retrieved, leading to a certain boundary beyond which UEFM scores cannot increase (i.e. 40)^18,20^. If CST integrity is beyond a certain level in a few weeks after the onset of stroke, the CST is generally used all the time, leading to high UEFM scores beyond 40^19,20^. Participants in the range below UEFM 20 are reasonably thought to use the CRST or any other alternative; while the CST looks to be activated after UEFM 20^4^. Hence it would be reasonable that 8 participants with UEFM 16 or below are assumed to preferentially use alternative tracts rather than the CST. For the range of UEFM between 20 and 40, there is no clear evidence in the literature on which tract is dominant. However our data showed that the measures of participants with UEFM between 20 and 40 are not significantly different from those with UEFM beyond 40. With these rationales, we could conclude that use of alternative tracts (i.e. CRST) results in a degradation in movement smoothness and slow activation of the MCP flexor.

Limitations of this study include the size of the sample. Although the small sample size leads to a barrier to generalization over the whole stroke population, we presented evidence that responses of flexors of the MCP joints following stroke depend on the degree of impairment. We reasoned that different neural motor pathways, which is hypothesized to be based on the degree of impairment, would lead to the dependency. Also we made inference based on UEFM scores regarding the dominance of tracts without support of imaging and neurophysiological studies. In the follow-up study, we will use imaging and neurophysiological approaches with a large sample to determine where differences in the neural mechanism for MCP flexion originate from.

## Data Availability

The data that support the findings of this study are available from the corresponding author on reasonable request.

## References

[1] Kamper DG, Rymer WZ (2001). Impairment of voluntary control of finger motion following stroke: Role of inappropriate muscle coactivation. Muscle Nerve.

[2] Raghavan P (2007). The nature of hand motor impairment after stroke and its treatment. Curr. Treat. Options Cardiovasc. Med..

[3] Wolbrecht ET, Rowe JB, Chan V, Ingemanson ML, Cramer SC, Reinkensmeyer DJ (2018). Finger strength, individuation, and their interaction: Relationship to hand function and corticospinal tract injury after stroke. Clin. Neurophysiol..

[4] Senesh MR, Barragan K, Reinkensmeyer DJ (2020). Rudimentary dexterity corresponds with reduced ability to move in synergy after stroke: Evidence of competition between corticoreticulospinal and corticospinal tracts?. Neurorehabil. Neural Repair.

[5] Barry AJ, Kamper DG, Stoykov ME, Triandafilou K, Roth E (2021). Characteristics of the severely impaired hand in survivors of stroke with chronic impairments. Top. Stroke Rehabil..

[6] Beer RF, Dewald JPA, Rymer WZ (2000). Deficits in the coordination of multijoint arm movements in patients with hemiparesis: Evidence for disturbed control of limb dynamics. Exp. Brain Res..

[7] Roh J, Rymer WZ, Perreault EJ, Yoo SB, Beer RF (2013). Alterations in upper limb muscle synergy structure in chronic stroke survivors. J. Neurophysiol..

[8] Wright ZA, Rymer WZ, Slutzky MW (2014). Reducing abnormal muscle coactivation after stroke using a myoelectric-computer interface: A pilot study. Neurorehabil. Neural Repair.

[9] Dewald JPA, Beer RF (2001). Abnormal joint torque patterns in the paretic upper limb of subjects with hemiparesis. Muscle Nerve.

[10] Owen M, Ingo C, Dewald JPA (2017). Upper extremity motor impairments and microstructural changes in bulbospinal pathways in chronic hemiparetic stroke. Front. Neurol..

[11] Wilkins KB, Owen M, Ingo C, Carmona C, Dewald JPA, Yao J (2017). Neural plasticity in moderate to severe chronic stroke following a device-assisted task-specific arm/hand intervention. Front. Neurol..

[12] Wilkins KB, Yao J, Owen M, Karbasforoushan H, Carmona C, Dewald JPA (2020). Limited capacity for ipsilateral secondary motor areas to support hand function post-stroke. J. Physiol..

[13] Baker SN (2011). The primate reticulospinal tract, hand function and functional recovery. J. Physiol..

[14] Schaechter JD (2009). Microstructural status of ipsilesional and contralesional corticospinal tract correlates with motor skill in chronic stroke patients. Hum. Brain Mapp..

[15] Karbasforoushan H, Cohen-Adad J, Dewald JPA (2019). Brainstem and spinal cord MRI identifies altered sensorimotor pathways post-stroke. Nat. Commun..

[16] Onodera S, Hicks TP (2010). Carbocyanine dye usage in demarcating boundaries of the aged human red nucleus. PLoS One.

[17] Jang SH, Lee SJ (2019). Corticoreticular tract in the human brain: A mini review. Front. Neurol..

[18] Schambra HM (2019). Differential poststroke motor recovery in an arm versus hand muscle in the absence of motor evoked potentials. Neurorehabil. Neural Repair.

[19] Senesh MR, Reinkensmeyer DJ (2019). Breaking proportional recovery after stroke. Neurorehabil. Neural Repair.

[20] Byblow WD, Stinear CM, Barber PA, Petoe MA, Ackerley SJ (2015). Proportional recovery after stroke depends on corticomotor integrity. Ann. Neurol..

[21] Prabhakaran S (2008). Inter-individual variability in the capacity for motor recovery after ischemic stroke. Neurorehabil. Neural Repair.

[22] Winters C, Van Wegen EEH, Daffertshofer A, Kwakkel G (2015). Generalizability of the proportional recovery model for the upper extremity after an ischemic stroke. Neurorehabil. Neural Repair.

[23] Balasubramanian S, Melendez-Calderon A, Burdet E (2012). A robust and sensitive metric for quantifying movement smoothness. IEEE Trans. Biomed. Eng..

[24] Vale FG, Cunha MJ, Schuch CP, Schifino GP, Balbinot G, Pagnussat AS (2021). Movement smoothness in chronic post-stroke individuals walking in an outdoor environment-A cross-sectional study using IMU sensors. PLoS One.

[25] Seo NJ, Rymer WZ, Kamper DG (2009). Delays in grip initiation and termination in persons with stroke: Effects of arm support and active muscle stretch exercise. J. Neurophysiol..

[26] Rohrer B (2002). Movement smoothness changes during stroke recovery. J. Neurosci..

[27] Chae J, Yang G, Park BK, Labatia I (2002). Delay in initiation and termination of muscle contraction, motor impairment, and physical disability in upper limb hemiparesis. Muscle Nerve.

[28] Beer RF, Dewald JPA, Dawson ML, Rymer WZ (2004). Target-dependent differences between free and constrained arm movements in chronic hemiparesis. Exp. Brain Res..

[29] Iwamuro BT, Cruz EG, Connelly LL, Fischer HC, Kamper DG (2008). Effect of a gravity-compensating orthosis on reaching after stroke: Evaluation of the therapy assistant WREX. Arch. Phys. Med. Rehabil..

[30] Maitland S, Baker SN (2021). Ipsilateral motor evoked potentials as a measure of the reticulospinal tract in age-related strength changes. Front. Aging Neurosci..

